# Iron Metabolism and Ferroptosis in Early Brain Injury after Subarachnoid Haemorrhage

**DOI:** 10.1007/s12035-024-04218-0

**Published:** 2024-05-23

**Authors:** Shihao Ge, Ziwen Jing, Lele Wang, Xiaocong Cui, Xin Zhang, Xiaopeng Wang

**Affiliations:** Department of Neurology, Second Hospital of Hebei Medical University, 215 Hepingxi Road, Shijiazhuang, 050000 Hebei China

**Keywords:** Subarachnoid haemorrhage, Early brain injury, Ferroptosis, Iron metabolism, Apoptosis, Neuroinflammation

## Abstract

At present, it appears that the prognosis for subarachnoid haemorrhage (SAH), which has a high death and disability rate, cannot be greatly improved by medication or other treatment. Recent research suggests that different types of cell death are implicated in early brain injury (EBI) after SAH, and this has been recognised as a major factor impacting the prognosis of SAH. Ferroptosis, which is a recently identified imbalance of iron metabolism and programmed cell death triggered by phospholipid peroxidation, has been shown to be involved in EBI after SAH and is thought to have a significant impact on EBI. The decomposition of cleaved haemoglobin during SAH involves the release of enormous amounts of free iron, resulting in iron metabolism disorders. Potential therapeutic targets for the signalling pathways of iron metabolism disorders and ferroptosis after SAH are constantly being discovered. To serve as a guide for research into other possible therapeutic targets, this paper will briefly describe the mechanisms of dysregulated iron metabolism and ferroptosis in the pathogenesis of SAH and highlight how they are involved in the development and promotion of EBI in SAH.

## Introduction

Subarachnoid haemorrhage (SAH) is a clinical syndrome with a variety of aetiologies, including blood vessel rupture at the base or surface of the brain, followed by blood flowing into the subarachnoid space. Its main cause is usually the rupture of an intracranial aneurysm. It has a significant death rate (35%) [1] and an incidence of 7.9 per 100,000 people [2], accounting for 5% of all strokes. The current treatment is still supportive care even though endovascular coils or neurosurgical aneurysm clipping can be used to repair a ruptured aneurysm [3]. Early brain injury (EBI), which occurs within 72 h of SAH, is now known to be a pathological process with substantial consequences that can increase mortality [4]. Multiple forms of injury have been reported to be involved in the EBI process, including oxidative stress, neuroinflammation, neurodegeneration, microcirculatory failure, excitotoxicity, blood–brain barrier (BBB) disruption, increased intracranial pressure, cerebral oedema, cell death and mitochondrial dysfunction [5]. Multiple types of neuronal death, including apoptosis, pyroptosis, autophagy, necrosis and ferroptosis, have been identified as being associated with EBI [3]. At present, the inhibition of iron death and iron metabolism through various methods has received widespread attention in the mechanism research of brain injury. In one study, haeme treatment reduced the survival of mice hippocampal neurons (HT-22) in culture by 50%, and haeme-induced neuronal cell death was comparable to ferroptosis brought on by erastin. Fer-1, a specialised inhibitor of ferroptosis, effectively reduced haeme-induced neuronal damage in contrast to targeted inhibitors of autophagy or apoptotic pathways. The results indicate the critical role ferroptosis plays in haeme-induced cell death in SAH-induced EBI [6].

Ferroptosis is a newly discovered non-apoptotic programmed cell death caused by the dysregulation of iron metabolism [7]. Numerous studies have discovered links between ferroptosis and a number of disorders, including cancers, kidney disease, neurological conditions like stroke, traumatic brain injury, Alzheimer's disease and Parkinson's disease [8–10]. Following SAH, haeme oxygenase 1 (HO-1)-catalysed haemoglobin released from lysed erythrocytes is further cleaved to haeme and phagocytosed by microglia/macrophages, which release significant amounts of free Fe^2+^. Because of the dysregulation of iron metabolism caused by these Fe^2+^ ions in the vicinity of the lesion, cell death, such as ferroptosis and apoptosis, is frequently observed [11]. Therefore, an important starting point in the disease course of EBI may be aberrant iron metabolism.

**Fig. 1:**
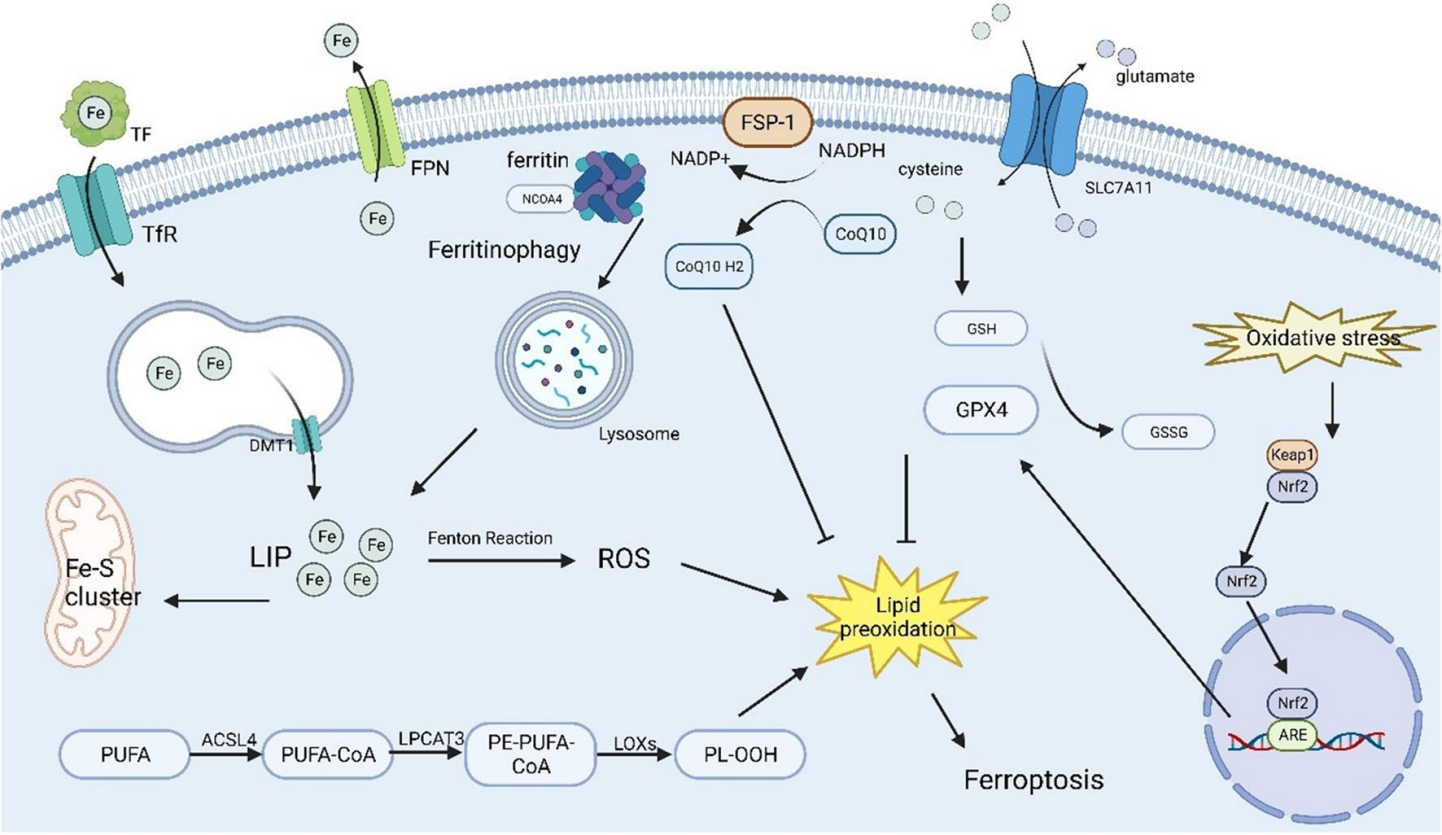
Potential therapeutic targets for the signaling pathways of iron metabolism disorders and ferroptosis after SAH. (The abbreviations: TF: transferrin; TfR: transferrin receptor; FPN: Ferroportin; FSP-1: fibroblast specific protein-1; CoQ10R: coenzyme Q10 receptor; SLC7A11: Recombinant Solute Carrier Family 7, Member 11; Nrf2: Nuclear factor erythroid 2-related factor 2GSSG; GPX4: glutathione peroxidase 4; LIP: labile iron pool; PUFA: polyunsaturated fatty acids; ROS: reactive oxygen species; PL-OOH: phospholipid hydroperoxides; PE: phosphatidylethanolamine; ACSL4: Acyl-CoA synthetase long-chain family; LPCAT3: lyso-phosphatidylcholine acyltransferase-3; LOX: low-density lipoprotein receptor; NADP: nicotinamide adenine dinucleotide phosphate; NADPH: nicotinamide adenine dinucleotide phosphate; DMT1: divalent metal transporter 1;NCOA4: nuclear receptor coactivator-4;GSH: glutathione)

Following this brief discussion of the mechanism of iron metabolism and ferroptosis, this paper now focuses on the functions of iron metabolism and ferroptosis in EBI after SAH and their prospective targets (Fig. 1). Related studies are listed in Table 1.

**Table 1:** Summary of the studies on iron accumulation and ferroptosis inhibition

Citation	Reagents	Models	Influence pathways	Main observation objects	Specific role
[3]	Sirtuin 1	In vivo: a mouse model of non-heparinized arterial blood injected into the anterior pool of the optic cross In vitro: HT22 cells	Enhance cellular antioxidant capacity; inhibit intracellular iron accumulation;	brain tissue	Up-regulation of GPX4 and FSP1 expression
^[[5]]^	L-NIL (inhibitor of iNOS)	Injection of autologous non-heparinized arterial blood into the anterior pool of the optic cross;	Inhibition of ferroptosis; reduction of neuroinflammation;	Neuronal cells; microglia;	Promotes ferroptosis in microglia of M1 phenotype; reduces neuroinflammation
^[[6]]^	Netin-1	In vivo: intravascular perforation model; In vitro: HT22 cells	PPARγ/Nrf2/GPX4 pathway and CoQ10-FSP1 pathway inhibit ferroptosis	brain tissue	Enhancement of PPARγ expression and further activation of Nrf2 expression;
^[[11]]^	Hepcidin Inhibitors	Injection of autologous non-heparinized arterial blood into the anterior pool of the optic cross;	Inhibition of iron accumulation;	brain tissue	Up-regulation of FPN1 and CP expression after SAH reduces intracellular iron accumulation
^[[16]]^	SC79 (Akt activator)	Injection of autologous non-heparinized arterial blood into the anterior pool of the optic cross;	Activation of the Akt/TfR/FPN1 pathway and inhibition of iron accumulation; Reduces lipid peroxidation	brain tissue	Reduces TfR expression; and promotes FPN1 expression; restores damaged Fe-S clusters and restores mitochondrial function
^[[24]]^	Quercetin	Endovascular perforation model	Enhancing intracellular antioxidant capacity;	Neuronal cells;	Up-regulation of GPX4, System XC-transport system and FPN1 expression; reduction of iron deposition
^[[25]]^	Heparin (Hepcidin inhibitor)	Endovascular perforation model	Inhibition of iron accumulation pathways and inhibition of ferroptosis;	brain tissue	Down-regulation of hepcidin and DMT1 expression and increase of FPN1 expression
^[[26]]^	Autophagy-associated gene 5 (ATG5) knockout	Endovascular perforation models;	Inhibition of autophagy onset and inhibition of iron accumulation;	brain tissue	Knockdown of ATG5 inhibits the autophagic signaling pathway and reduces the release of free iron ions from the autophagic breakdown of ferritin;
^[[27]]^	Baicalin	Optic cross anterior pool injection of autologous arterial blood model	Inhibition of autophagy-dependent ferroptosis; inhibition of neuronal apoptosis; inhibition of autophagic signaling in brain tissue	brain tissue	Inhibition of autophagy-dependent ferroptosis
^[[28]]^	Inhibition of S100A8 expression	Endovascular perforation model	Inhibition of autophagy-dependent ferroptosis in microglia and inhibition of iron accumulation;	Microglia;	Inhibition of NCOA4-mediated autophagy in microglia and reduction of iron ion release;
^[[30]]^	Deferoxamine	Injection of autologous non-heparinized arterial blood into the anterior pool of the optic cross;	Inhibition of iron accumulation;	Brain tissue; microglia	Increase the expression level of HO-1 in microglia and macrophages
^[[33]]^	pifithrin-α (p53 inhibitor)	Endovascular perforation models;	Inhibition of ferroptosis through inhibition of p53 expression;	Rat cerebral cortex cells;	Inhibition of p53 expression
^[[31]]^	Fer-1	In vivo: intravascular perforation model; In vitro: SH-SY5Y hemoglobin-treated cells;	Inhibition of lipid peroxidation; inhibition of iron accumulation;	brain tissue	Up-regulation of FPN expression; reduction of iron content; improvement of lipid peroxidation;
^[[32]]^	Liproxstatin-1	In vivo: intravascular perforation model In vitro: HT22 cells	Inhibits ferroptosis; inhibits microglia activation and neuroinflammation	Neuronal cells; microglia;	Stabilize the expression of GPX4; down-regulate the expression of ACSL4 and COX2
^[[34]]^	Astragaloside	Endovascular perforation;	Nrf2/HO-1 access	brain tissue	Promoting nuclear translocation of Nrf2 from the cytoplasm to the nucleus;
^[[35]]^	Puerarin	A modified model of endovascular perforation;	AMPK/PGC1/Nrf2 pathway inhibits ferroptosis; ameliorates oxidative stress;	brain tissue	Activation of Nrf2 via AMPK/PGC1/Nrf2 pathway to stabilize intracellular antioxidant system
^[[39]]^	Cepharanthine (ALOX15 inhibitor)	In vivo: intravascular perforation model; In vitro: mouse BV2 lineage microglia; bEnd.3 microvascular endothelial cells;	Inhibition of lipid peroxidation pathway;	Microglia; vascular endothelial cells;	Down-regulation of ALOX15 expression
^[[40]]^	AQP4 overexpression	Endovascular perforation models;	Inhibition of transferrin infiltration further attenuates iron accumulation;	brain tissue	Inhibition of transferrin infiltration into the lymphatic system
^[[51]]^	Protein kinase R inhibition	Endovascular perforation models;	Inhibition of the ferroptosis pathway; endoplasmic reticulum emergency pathway correlates;	brain tissue	Inhibition of protein kinase R expression and suppression of ferroptosis occurrence

The abbreviations: AMPK: Adenosine 5 ‘-monophosphate (AMP)-activated protein kinase; PGC1: peroxisome proliferator-activated receptor gamma coactivator 1; Nrf2: Nuclear factor erythroid 2-related factor 2; AQP4: Aquaporin-4; HT22: heme-treated mice hippocampal neurons; PPARγ: Peroxisome proliferator-activated receptor gamma; GPX4: glutathione peroxidase 4; CoQ10-FSP1: coenzyme Q10-ferroptosis suppressor protein 1; NCOA4: Nuclear receptor coactivator 4; FPN1: Ferroportin1; L-NIL: l- N(6)-(1-iminoethyl)lysine; iNOS: Inducible nitric oxide synthase; ALOX15: arachidonic acid 15-lipoxygenase-1; BV2: Mouse Microglia Cells; ACSL4: Acyl-CoA synthetase long-chain family; COX2:Cyclooxygenase 2; SH-SY5Y: neuroblastoma; DMT1: divalent metal transporter 1; SC79: AKT activator; Akt/TfR/: V-akt murine thymoma viral oncogene homolog; TfR: transferrin receptor; CP: ceruloplasmin; SAH: Subarachnoid hemorrhage; ATG5: autophagy related 5

## Iron Metabolism in Early Brain Injury after Subarachnoid Haemorrhage

Iron is the most prevalent mineral in the brain and a crucial enzyme cofactor in numerous biological processes. It is involved in crucial pathways that include cellular respiration and the synthesis of myelin [12]. Since iron deficiency and iron overload can both lead to the development of various diseases, it is important to investigate and understand the controlling factors of iron metabolism.

Through nutrition, iron is absorbed mostly as Fe3 + , which binds to transferrin and the transferrin receptor 1 (TfR1) on cell membranes in the peripheral blood, enters the cells through endocytosis to form endosomes and is then reduced to Fe^2+^. Upon entry into the cell, divalent metal transporter protein (DMT1) transports iron from the endosome to the cytoplasm [13]. Intracellular Fe^2+^ can be stored in the cell as ferritin or pumped out of the cell by the membrane protein ferroportin (FPN). While the majority of this iron is transported to the mitochondria for the formation of haeme or Fe-S clusters, intracellular iron forms a labile iron pool (LIP) [14]. To maintain normal mitochondrial function, both iron and Fe-S clusters can be involved in the synthesis of lipid peroxide-producing enzyme systems, such as arachidonate lipoxygenase (ALOX), nicotinamide adenine dinucleotide phosphate hydrogen oxidase and other iron-containing enzymes.

After SAH, neuronal cells will be exposed to abnormally high concentrations of haeme. Recent studies in mouse models have demonstrated that both transferrin (Tf) and TfR levels are elevated in brain tissue after the onset of SAH, and significant increases in non-haeme iron and ferritin levels were observed in the brains of mice after SAH, suggesting that iron accumulation occurs in the brain parenchyma after SAH [3,15,16]. Through the Tf–TfR pathway and ferritin phagocytosis, more iron ions enter the cells. The intracellular LIP increases when intracellular ferritin is unable to store extra iron ions. As a result of the Fenton reaction, the Fe^2+^ produces extremely dangerous hydroxyl radicals when it comes into contact with hydrogen peroxide (H_2_O_2_) [14]. This encourages intracellular lipid peroxidation, causing the peroxidation of unsaturated fatty acids in the cell membrane, which ultimately results in ferroptosis.

## Ferroptosis in Early Brain Injury after Subarachnoid Haemorrhage

In 2012, Dolma et al. made the discovery of a novel chemical called erastin while evaluating potential anticancer medications. No signs of chromatin condensation or margination, deoxyribonucleic acid (DNA) fragmentation, activation of caspases (the mediator of apoptosis and pyroptosis), swelling of the cytoplasm or organelles that appear during necrosis or double-membrane vesicles produced by cellular autophagy were observed in erastin-induced cell death [17]. The concept of ferroptosis was formally introduced by Dixon et al. in 2012 after several years of intensive research on this recently discovered type of death, and it was demonstrated that this process is characterised by the accumulation of large amounts of iron-dependent lipid reactive oxygen species (ROS) that cause cell death [7]. Three key characteristics of ferroptosis are the inability of lipid peroxide to be scavenged, the presence of redox-active iron, and the oxidation of phospholipids containing polyunsaturated fatty acids (PUFA) [18]. Morphologically, intact cell membranes, cell swelling, mitochondrial shrinkage and increased mitochondrial membrane density, reduction or loss of mitochondrial cristae without chromatin condensation or marginal clustering are observed in ferroptotic cells [17,19]. Biochemically, excessive lipid peroxides cannot be reduced, and ROS accumulate in large amounts on membrane lipids, impairing the original biological functions of the cell membrane and, ultimately, resulting in cell death. This is accompanied by the depletion of intracellular glutathione (GSH) and loss of glutathione peroxidase 4 (GPX4) activity.

GPX4 is a crucial enzyme in preventing ferroptosis [20]. It uses GSH as a cofactor to convert lipid peroxides into non-toxic lipid alcohols. GSH, a major cellular antioxidant, is essential for GPX4's activity. When GPX4 function is intact, it prevents the accumulation of lipid peroxides, thus inhibiting ferroptosis. Lipid peroxidation is a key event in the initiation of ferroptosis. It involves the oxidative degradation of polyunsaturated fatty acids within cell membranes, leading to the formation of lipid peroxides. These lipid peroxides are highly reactive and can cause extensive cellular damage if not reduced by GPX4. When the activity of GPX4 is compromised or when GSH levels are depleted (e.g., due to insufficient synthesis or excessive consumption), lipid peroxides accumulate unchecked. This accumulation is the direct cause of the oxidative damage to cellular membranes seen in ferroptosis. Thus, the GPX4/GSH axis is central to the regulation of ferroptosis, with its ability to control lipid peroxide levels being a determinant of cell survival or death.

Lipid peroxidation (LPO) is regarded as a marker of the ferroptosis mode of cell death [21]. Toxic hydroxyl radicals that build up in the cell membrane and cause alterations to its structure and function, as well as disruptions to normal cell metabolism, are the primary catalysts for LPO. Inhibition of the aforementioned pathways results in insufficient GSH content, decreased GPX4 activity, or excessive lipid peroxide production that exceeds the antioxidant capacity of the cell, leading to the buildup of lipid peroxides inside the cell and the breakdown of the membrane lipid structure, which causes ferroptosis [14,22]. GPX4 and GSH are currently a hot research area since they are so important in preventing ferroptosis.

## Potential Therapeutic Targets for Ferroptosis in Early Brain Injury after Subarachnoid Haemorrhage

### Inhibition of Iron Accumulation

Through the Fenton reaction, more Fe^2+^ produces extra hydroxyl radicals, aggravating intracellular oxidative stress and raising intracellular ROS levels. The overproduction of ROS weakens Fe-S clusters inside mitochondria, resulting in dysfunctional mitochondria, and triggers ferroptosis. Lee initially researched the effect of iron in early brain damage following SAH. Deferoxamine (DFX), an iron chelator, was reported to decrease mortality, oxidative DNA damage and the induction of iron-treated proteins [23]. In a study employing SC79 (an Akt activator, whose activation suppresses intracellular oxidative stress by upregulating SOD1/2), iron levels in brain tissue after SAH were lower, as were ROS and MDA levels, and the activity of injured Fe-S clusters was restored [16]. Quercetin has also been found to reduce iron deposition and lipid peroxidation after SAH, but the exact mechanism of action is not yet understood [24]. Overall, iron chelators appear to have a clear role in suppressing ferroptosis and improving SAH outcomes. It is necessary to find compounds with fewer side effects and more stable properties and to discover which mode of administration can produce the best drug effects to accelerate the transition to clinical application. Ceruloplasmin (CP), which is present in cerebrospinal fluid, is a ferrous oxidase that can be synthesised and secreted by brain tissues. It can promote the oxidation of ferrous iron to trivalent iron and binding to transferrin, which plays an important role in the iron transport system and iron homeostasis [11]. Hepcidin is an iron-regulating protein that can inhibit FPN and CP expression in the rat cerebral cortex and hippocampus, promoting DMT1 expression and increasing iron accumulation [25]. In the SAH rat model, the expression levels of hepcidin and DMT1 were increased, while the downstream targets FPN and CP showed lower expression levels [11,25]. After treatment with small interfering ribonucleic acid targeting hepcidin, the restoration of FPN and CP levels was observed, while a decrease in apoptosis levels could be observed with transferase uridyl nick end labelling staining [11]. Additional research confirmed that hepcidin might similarly stimulate the production of DMT1, raise intracellular amounts of free iron ions and trigger ferroptosis. This implies that DMT1 may play a significant regulatory role in the development of ferroptosis in EBI. Evidence suggests that the lysosomal autophagic pathway is essential for ferritin degradation and iron release [12]. The incidence of ferroptosis is therefore made worse by the occurrence of ferritinophagy, which is the specific autophagic pathway that uses ferritin as a degradation substrate and, seemingly, an autophagic cell death pathway [26]. One study found that after SAH, the expression level of the autophagy marker LC3II peaked 24 h later. From 6 to 48 h, the expression of ferritin heavy chain 1 (FTH1) began to decline, indicating that FTH1 had begun to degrade, releasing free iron ions and causing ferroptosis. Additionally, the autophagic pathway was suppressed by the targeted silencing of the autophagy-related gene 5 (ATG5), which decreased ferritin degradation and restored GPX4 activity [26]. Another study found that the application of baicalin intraperitoneal injection therapy was effective in lowering the LC3-II/LC3-I ratio after SAH, reducing the occurrence of autophagy and alleviating EBI [27]. Targeted inhibition of S100A8 gene expression in microglia was able to inhibit the expression of nuclear receptor coactivator-4 (NCOA4), which, in turn, suppressed the occurrence of NCOA4-mediated ferritinophagy and reduced the release of free iron ions, preventing ferroptosis [28]. The above findings suggest that autophagy mediates the occurrence of ferroptosis. Ferritin may be triggered in some way to enhance more ferritinophagy because it is unable to store additional iron ions due to the excess iron content, which could cause further ferroptosis. Further research is required to elucidate the precise mechanism, as it may be crucial in preventing the development of EBI following SAH.

Haemopexin (Hpx) is a plasma protein that is widely expressed and produced by glial cells and neurons [29]. One study found that it counteracts the redox toxicity of haeme by forming haeme-Hpx complexes with haeme and facilitating absorption by low density lipoprotein receptor-related protein 1 (CD91)-positive cells, preventing further breakdown to free iron ions and free radical reactions [12]. The Hpx scavenging system was significantly active after SAH, but the study also revealed that patients with high levels of Hpx in the cerebrospinal fluid had a worse prognosis. This is likely because Hpx binds to haeme, changes its molecular size and prevents the brain from excreting excess haeme, and the haeme overload then causes additional damage [29]. The mechanism of the Hpx-CD91 clearance system needs to be explored further to discover potential therapeutic targets.

Two previous studies noted a significant increase in HO-1 co-localisation of cortical microglia expression in a mouse model after SAH. In Lee's study, where mice were treated with an intraperitoneal administration of DFX, a significant decrease in HO-1 expression levels in the basal cerebral cortex region was observed [15]. However, in LeBlanc's study, treating mice with an intracerebroventricular injection of DFX significantly increased the expression level of HO-1 in microglia while concurrent intraperitoneal administration showed no significant increase in microglia HO-1 levels. It was also observed that the elevation of HO-1 levels in bone marrow-derived cells (including leukocytes, microglia and granulocytes) after DFX treatment produced neuroprotective effects [30]. The two studies suggest that different modes of administration can influence how DFX works and that changes in HO-1 after DFX treatment of different cell types vary, presenting new ideas for further experimental validation as well as clinical practice.

### Enhancement of Cellular Antioxidant Capacity

Numerous studies have shown that GPX4 begins to decrease within 24 h of SAH [26,31,32] and the expression of cystine transporter solute carrier family 7 member 11 (SLC7A11) is also noticeably lowered [33], accompanied by a decrease in GSH levels in cortical tissue. Increased GPX4 expression can improve mice's neurobehavioral abilities, lower levels of lipid peroxidation following experimental SAH and prevent neuronal death and brain oedema. The application of ferroptosis inhibitors, such as Fer-1 and Lip-1, can promote GPX4 expression and increase GSH levels in mice [32]. The upregulation of Nrf2 expression with the administration of puerarin and astragaloside stabilised the expression of GPX4 in the intracellular antioxidant system and attenuated oxidative stress and ferroptosis [6,34,35]. Netin-1 and epigenetic factor Sirtuin 1 elevated the expression level of GPX4 and activated the FSP1-CoQ10-NADPH pathway, which together increased the intracellular antioxidant capacity [3,6]. Since ML385 (an HO-1 inhibitor) reversed astragaloside's protective effect, it is possible that the Nrf2/HO-1 pathway plays a more significant role in preventing ferroptosis after SAH [34]. Ferroptosis in EBI may be partially dependent on p53 activation, as evidenced by the fact that using pifithrin-α to specifically block the production of the p53 gene can greatly enhance the expression of SLC7A11 and GPX4 and diminish the occurrence of ferroptosis following SAH [33].

In conclusion, a number of preclinical investigations have demonstrated the protective benefits of ferroptosis suppression and the stability of intracellular antioxidant capacity at several levels of the regulatory sites of the ferroptosis pathway. It would appear that GPX4 activity is a cornerstone of intracellular antioxidant defence and could be a key regulatory target for blocking ferroptosis from further lipid peroxidation. Studies have also reported that the enhancement of GPX4 activity represents the enhancement of cellular antioxidant capacity and reduces the occurrence of nerve damage, and a variety of herbal components, due to their own beneficial antioxidant effects, can enhance the antioxidant capacity of cells through various signalling pathways, reducing the occurrence of oxidative stress and ferroptosis after SAH. Moreover, the broader effects of traditional Chinese medicine ingredients, such as their anti-inflammatory and anti-apoptotic effects, need further research to clarify their mechanisms and make them more effective.

### Inhibition of Lipid Peroxidation

Lipid peroxidation is the process by which oxidants attack the carbon–carbon double bonds of lipids, particularly PUFA [36]. Lipid peroxidation mostly occurs in cell membranes, where leading to structural and functional alterations that have a negative impact on cell metabolism and operation. Neuronal cell membranes, which are rich in polyunsaturated fatty acids (PUFAs), are particularly vulnerable to lipid peroxidation. PUFAs such as arachidonic acid (AA) and adrenic acid (AdA) undergo a process where they are first converted by acyl coenzyme A synthase (ACSL4) into AA/AdA-CoA. This compound is then further processed by Lys phosphatidylcholine acyltransferase 3 (LPCAT3), resulting in the formation of reactive oxygen species (ROS) precursors, including PE-AA and PE-AdA, which contribute to cell death [36]. Malondialdehyde (MDA) and 4-hydroxy-nonenal (4-HNE) are hazardous lipid hydroperoxides and aldehydes that the direct oxidation of lipoxygenase (LOX) eventually produces, and they have long been utilised as indicators of ferroptosis [37]. ACSL4 and LPCAT3 serve as the rate-limiting components in the synthesis of PE-AA and PE-AdA and are also essential regulatory factors for the onset of ferroptosis [36].

The expression of ACSL4 was markedly increased after SAH and peaked on day 3 [3,32]. Lip-1 treatment was able to lower the expression of ACSL4 and its metabolite, 5-hydroxyeicosapentaenoic acid (5-HETE). However, the higher levels of 5-HETE subsequently recurred, suggesting the existence of other mechanisms of accumulation [38]. Another study showed that the level of ALOX15 remained high for a period of 24 to 72 h after SAH and was mainly expressed in the cortical M2 microglia near the blood clot and in the distal cortical endothelial cells away from the blood clot. The use of cepharanthine, an alkaloid extracted from Chinese herbal medicine, significantly inhibited the expression level of ALOX15 in microglia as well as endothelial cells, thereby inhibiting the level of lipid peroxidation [39].

In summary, lipid peroxidation is a key process of ferroptosis. Existing studies have found that inhibiting key enzymes in the lipid peroxidation signalling pathway can effectively reduce ferroptosis. At the same time, it is suggested that ferroptosis not only exists in neurons but can also affect microglia and endothelial cells. This possibility lays the foundation for further research on how ferroptosis plays a role in EBI. Moreover, ferroptosis in different types of cells (such as M1 or M2 microglia) has different effects on the development of EBI. Therefore, whether the targeted regulation of certain types of cell ferroptosis would be effective also needs further exploration.

### Reduction of Blood–Brain Barrier Disruption

After SAH, the integrety BBB continues to be compromised during EBI, and protein leakage into the brain parenchyma increases significantly, exacerbating cerebral edema and raising intracranial pressure in addition to brain ischemia and hypoxia [32,33]. Treatment with Fer-1 and Lip, a ferroptosis-specific inhibitor, drastically decreased protein leakage and downregulated MMP-9 expression in the mouse model [32,33]. Another study discovered that transferrin leakage dramatically increased after SAH and that perivascular aquaporin 4 (AQP4) expression was significantly lowered. In contrast, synthetic AQP4 overexpression decreased astrocyte AQP4 polarisation in the BBB, stabilised the BBB 's operation and lowered the amount of transferrin found in the brain parenchyma, which decreased the incidence of ferroptosis. Reduced iron access into the brain parenchyma also indirectly decreased the amount of ferritin there, which decreased the occurrence of ferroptosis and enhanced neurological function [40]. Additionally, when the BBB function is stabilised, ferroptosis does not appear to occur. However, the specific process requires further investigation.

### Inhibition of Inflammatory Processes

It is known that M1 phenotype microglia release the pro-inflammatory molecule inducible nitric oxide synthase (iNOS), thereby increasing their resistance to ferroptosis. It has been demonstrated that M1 phenotype microglia quickly multiply after SAH, generating a localised pro-inflammatory milieu and harming neurons [5]. While directly lowering their pro-inflammatory effect, the use of iNOS inhibitors can induce ferroptosis in M1 phenotype microglia and work in concert to lessen the inflammatory response following SAH [5]. One study found that the number of CD68/Iba-1 positive microglia increased considerably after SAH but decreased significantly after a Lip-1 injection. The levels of SAH-induced pro-inflammatory factors were significantly upregulated at three days after injury, and Lip-1 and Fer-1 prevented the increased expression of pro-inflammatory factors, such as interleukin 6 (IL-6), tumour necrosis factor alpha and IL-1b [32,33]. These findings suggest that inflammation and ferroptosis in EBI may have mutually promoting effects and that other forms of death in EBI, such as necrosis, apoptosis and pyroptosis, may also have pro-inflammatory effects. The specific cascade reaction has not yet been elucidated, and more research is needed to verify whether the various forms of death have a synergistic effect on inflammation.

### Activating the Ferroptosis Suppressor Protein-Coenzyme Q10-Nicotinamide Adenine Dinucleotide Phosphate Pathway

When the ferroptosis suppressor protein (FSP1), also known as apoptosis-inducing factor mitochondria-associated 2 (AIFM2), was found, it was not believed to have anti-ferroptotic properties [41,42]. However, more recent studies have revealed that FSP1 has nicotinamide adenine dinucleotide phosphate** (**NADH)-ubiquinone oxidoreductase activity that can reduce coenzyme Q10 (CoQ10) to the corresponding ubiquinol form (CoQ10H2). As an essential antioxidant, CoQ_10_H_2_ can eliminate free radicals and lessen oxidative stress, and it plays an important role as a reversible redox carrier in electron transport across the plasma membrane and the Golgi membrane of cells. It also acts as an endogenous lipid-soluble antioxidant by directly scavenging lipid peroxyl radicals. This process can inhibit the propagation of lipid peroxides, thereby preventing ferroptosis. This pathway acts independently of GSH/GPX4 to inhibit ferroptosis and phospholipid peroxidation [6], and it may play an important anti-ferroptosis role in synergy with GPX4 in EBI following SAH.

### Activating the Keap1-Nuclear Factor Erythroid-2-Related Factor 2/antioxidant Response Element Nuclear Translocation Pathway

Keap1 protein is an important intracellular antioxidant defence protein. It binds to nuclear factor erythroid-2-related factor 2 (Nrf2) in the cytoplasm under non-stress conditions and mediates the degradation of Nrf2, a transcriptional factor, through ubiquitin labelling. After being stimulated by stress, Nrf2 will dissociate from Keap1, go into the nucleus, bind to the antioxidant response element (ARE) and begin to accumulate there [34]. It regulates the expression of more than 250 genes by binding to ARE sites [43], and, to prevent the onset of ferroptosis, Nrf2 modulates the expression of GCL and GS and upregulates the expression of GPX4, SLC7A11 and FTH1 [44]. Furthermore, HO-1, a downstream regulatory gene of Nrf2, has cytoprotective and antioxidant capabilities. It has been demonstrated that HO-1 overexpression lowers ferroptosis and guards against irreversible kidney damage [45]. Additionally, Nrf2 activation decreases cellular iron uptake, boosts iron storage and reduces ROS production, eventually controlling glutathione synthesis, lipid peroxidation and iron metabolism.

### Inhibition of p53 Expression

The tumour suppressor p53 serves as a major regulator of apoptosis. The main function of p53 is to mediate cellular and systemic metabolism, and recent studies have found that p53 is closely associated with the metabolic pathways involved in ferroptosis, which it is thought to promote [46]. First, p53 can inhibit the transcription of SLC7A11 and reduce the function of the system XC-transport system, by interfering with cystine uptake, reducing GSH synthesis and decreasing the cellular antioxidant capacity. At the same time, p53 has been found to promote the activity of arachidonic acid 12-lipoxygenase (ALOX12), a pathway that is thought to act independently of GPX4 in targeting ferroptosis and promoting its occurrence [47]. Secondly, the rate-limiting enzyme in polyamine catabolism, called spermidine/spermine N1-acetyltransferase 1 (SAT1), is activated by p53 and also causes the overexpression of arachidonate 15-lipoxygenase (ALOX15), which promotes lipid peroxidation and causes ferroptosis [46]. Additionally, it has been demonstrated that p53 can promote ferroptosis [48], induce aberrant redox iron buildup and perform its functions independently of GPX4 [49]. In conclusion, new p53-related mechanisms in ferroptosis regulation are being uncovered, and most of these studies have demonstrated that inhibiting p53 expression prevents the occurrence of ferroptosis.

## Discussion and Conclusion

Research has recently turned its focused-on EBI after SAH, and as the investigation into aberrant iron metabolism in EBI has progressed, it has been discovered to be strongly related to a recently identified type of cell death known as ferroptosis. Its main characteristics are the dysregulation of the antioxidant system, the disruption of iron ion metabolism and the buildup of lipid peroxides. It should be noted that unlike intraparenchymal haemorrhage, the SAH begins with an abnormal flow of blood into the subarachnoid space. During the development of SAH, the effective removal of abnormally increased haemoglobin in the cerebrospinal fluid of the subarachnoid space can effectively block further damage to the brain parenchyma. However, the mechanism of the Hpx-CD91 clearance system remains unclear and needs to be further explored. Meanwhile, more targets need to be discovered in the future to maintain the stability of the BBB in the early stages of SAH and to reduce the leakage of blood haemoglobin and transferrin into the brain parenchyma. LeBlanc's study suggested that for SAH, different modes of administration may have a significant impact on outcomes [30], and further comparative studies are needed. In addition, the intraventricular administration of drains in high-grade SAH patients is now possible and needs to be further investigated. According to the current data, haeme-induced ferroptosis is a type of classical ferroptosis that does not depend on the Ras-Raf-MEK-ERK pathway [50]. In the context of hemorrhagic stroke, studies have demonstrated that NTN-1 improves neurological outcomes in mice and protects neurons from death caused by neuronal ferroptosis. Furthermore, the mechanism underlying NTN-1 neuroprotection is correlated with the inhibition of ferroptosis, attenuating cell death via the PPARγ/Nrf2/GPX4 pathway and coenzyme Q10-ferroptosis suppressor protein 1 (CoQ10-FSP1) pathway[6]. Additionally, NAC can effectively alleviate neuronal cell death and promote functional recovery in rat ICH models by neutralizing lipid peroxidation produced by ALOXs [24,51]. However, this still needs to be confirmed and defined in terms of EBI harm.

In addition, the increase in transferrin and TfR levels in brain tissue did not occur immediately but was observed after the onset of subarachnoid hemorrhage (SAH). According to this study, significant increases in non-haeme iron and ferritin levels, which are indicative of iron accumulation that would involve Tf and TfR pathways, were noted in the brains of mice after SAH. This suggests that the rise in Tf and TfR levels likely developed as part of the response to iron dysregulation following the initial haemorrhage event. The relationship between ferroptosis and other injuries also deserves further exploration. Inhibitors that focus on a specific type of cell death have not yet shown a meaningful advantage in clinical studies. Therefore, the relationship between ferroptosis and the occurrence of other forms of injury, such as autophagy, apoptosis, and inflammation, must be investigated further to discover more potential targets for combination therapy.

In conclusion, research into abnormal iron metabolism and the occurrence of ferroptosis in EBI is still in its early stages. Numerous potential therapeutic targets still require investigation, including improved drug delivery systems, and a quicker turnaround to clinical trials is essential.

## Data Availability

All data generated or analyzed during this study are included in this published article.
